# Supplementary material for: Compound C Inhibits B16-F1 Tumor Growth in a Syngeneic Mouse Model via the Blockage of Cell Cycle Progression and Angiogenesis
Source: Cancers (Basel). 2019 Jun 13;11(6):823. doi: 10.3390/cancers11060823 (PMC6627246; doi:10.3390/cancers11060823)
Supplement: Supplementary file 1 [file cancers-11-00823-s001.pdf]

Supplementary Materials: Compound C Inhibits B16-F1 Tumor Growth in a Syngeneic Mouse Model Via the Blockage of Cell Cycle Progression and Angiogenesis

Yun Taek Lee, So Hyun Lim, Boram Lee, Insug Kang and Eui-Ju Yeo

S1-1

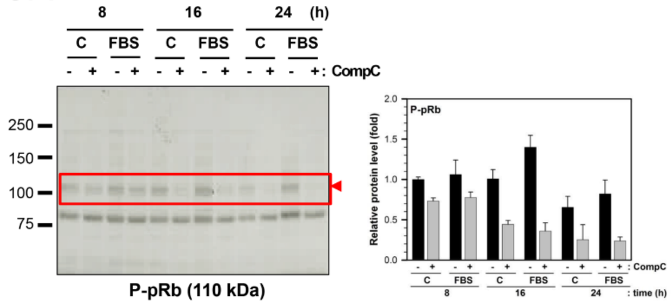

S1-2

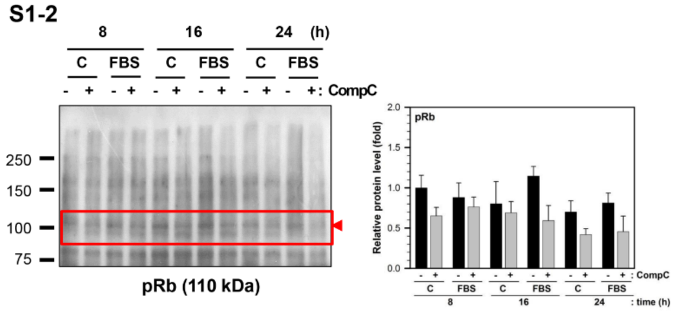

S1-3

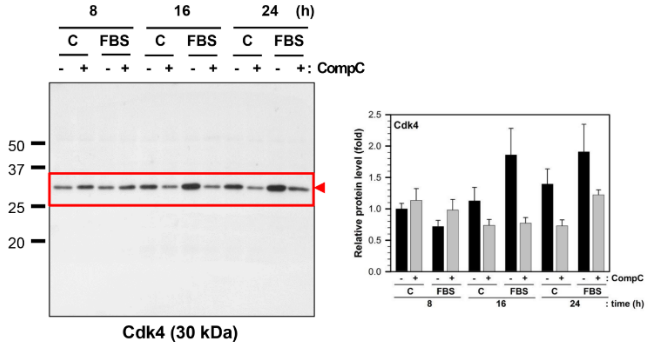

S1-4

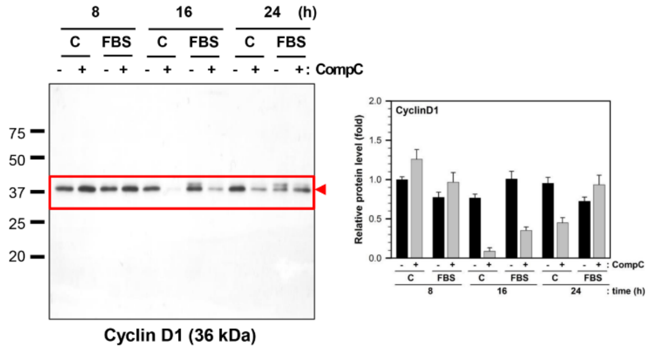

S1-5

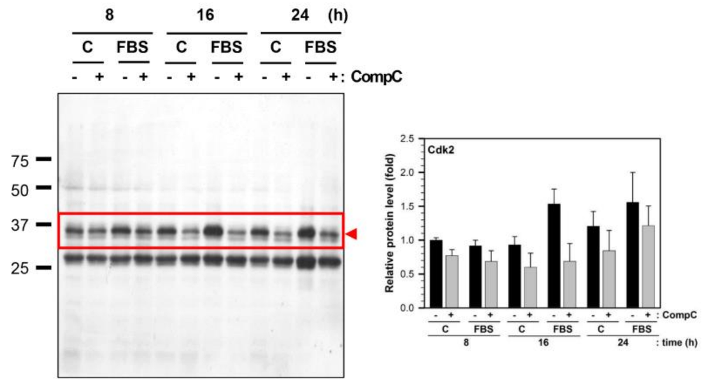

Cdk2 (33 kDa)

S1-6

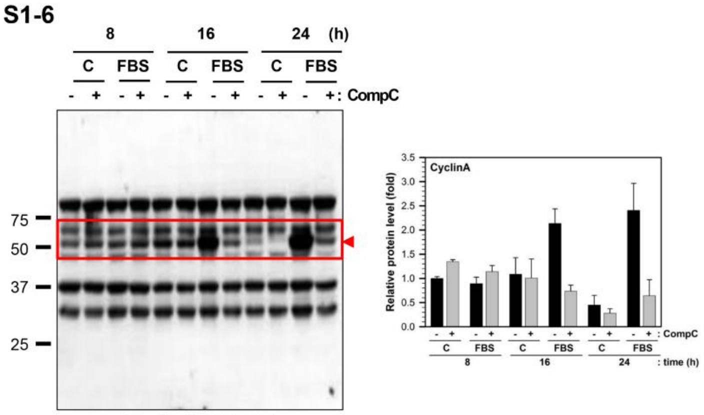

Cyclin A (55 kDa)

S1-7

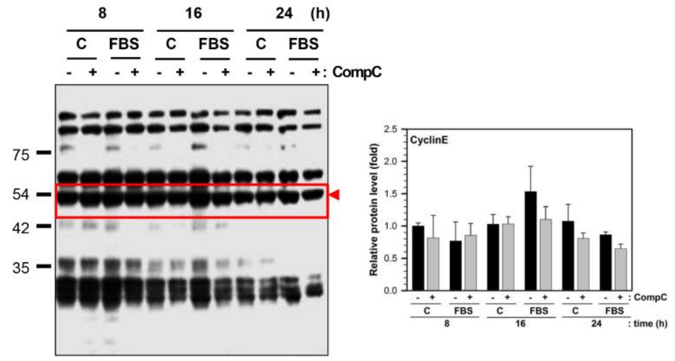

Cyclin E (48 kDa)

S1-8

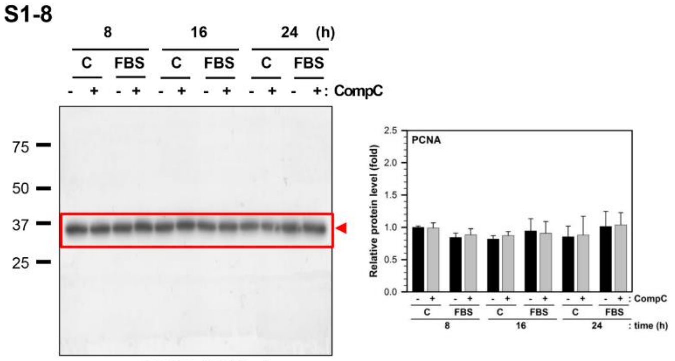

PCNA (36 kDa)

S1-9

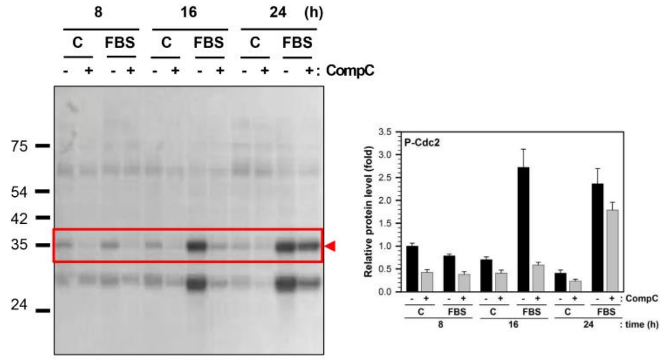

P-Cdc2 (34 kDa)

S1-10

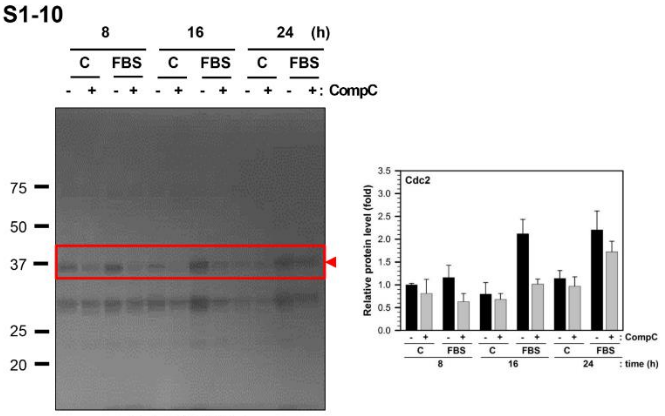

Cdc2 (34 kDa)

S1-11

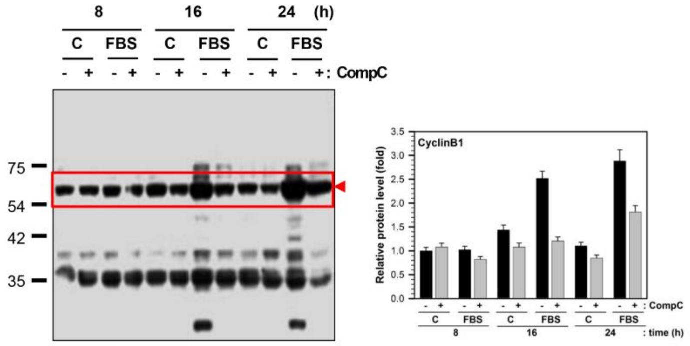

Cyclin B1 (55 kDa)

S1-12

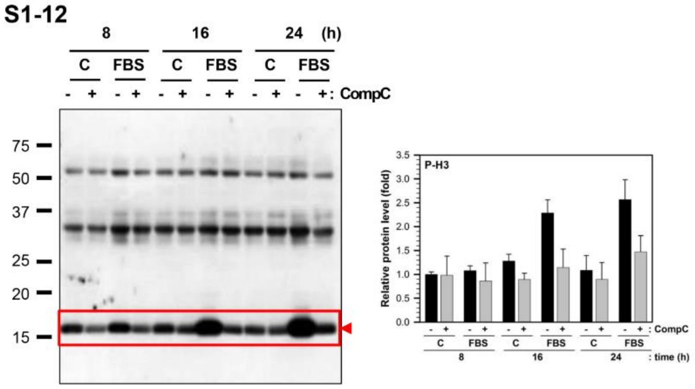

P-H3 (17 kDa)

S1-13

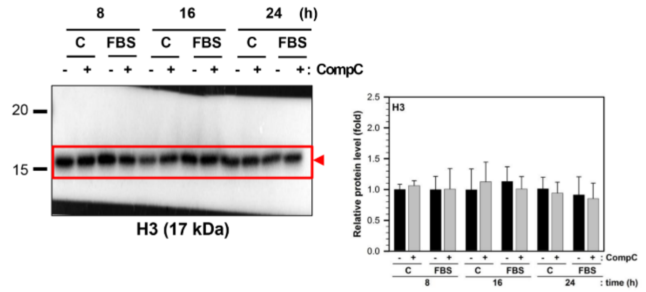

S1-14

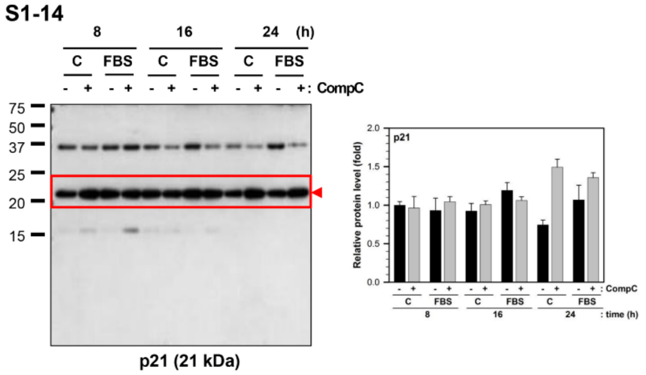

S1-15

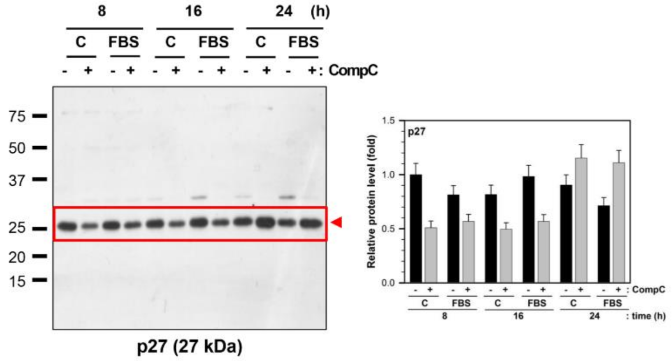

S1-16

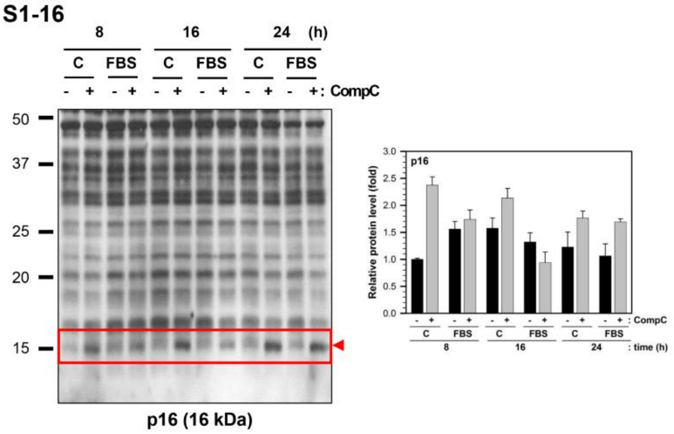

S1-17

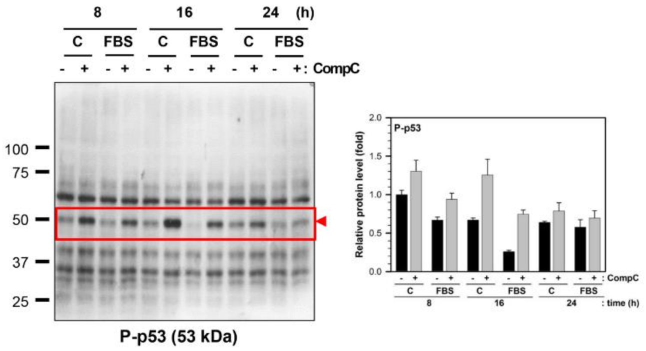

S1-18

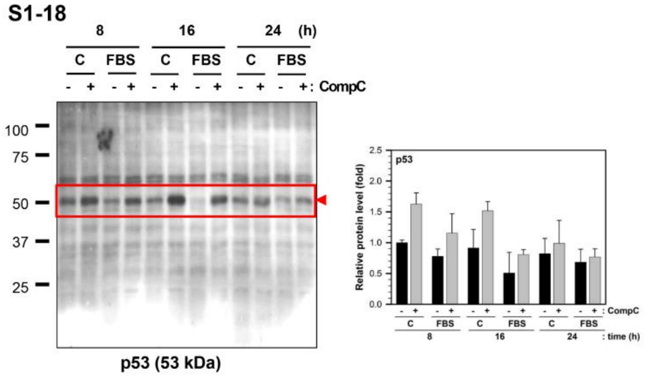

S1-19

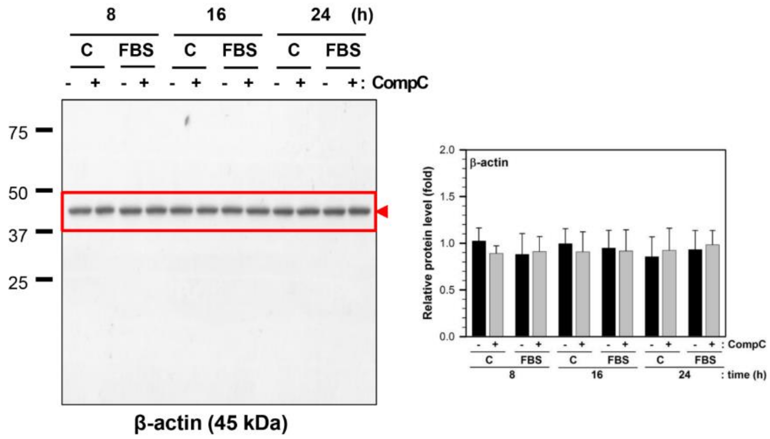

Figure S1. Quiescent cells were stimulated with 10% FBS for the indicated times (8, 16, and 24 h) in the presence of vehicle (−) or 10 μM CompC (+). Cell lysates were analyzed by western blot analysis using antibodies against total and phosphorylated pRb (P-pRb), Cdks, cyclins, histone H3, Cdk inhibitors, and β-actin as an internal control. The band density of β-actin was used as a normalization control for densitometry analysis of each blot. A representative whole blot and a graph with error bars for western blot analysis of each protein are shown side by side. (supplementary data for Figure 2A,B)

S2-1

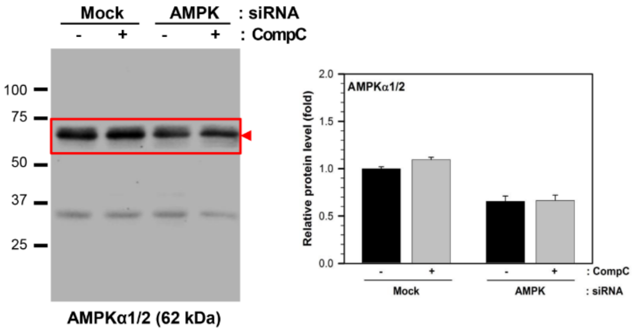

S2-2

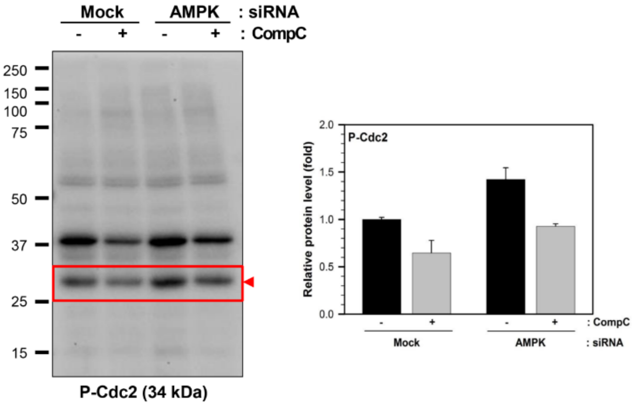

S2-3

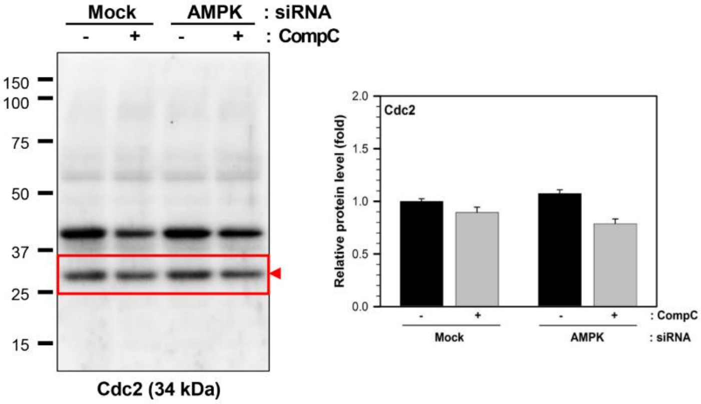

S2-4

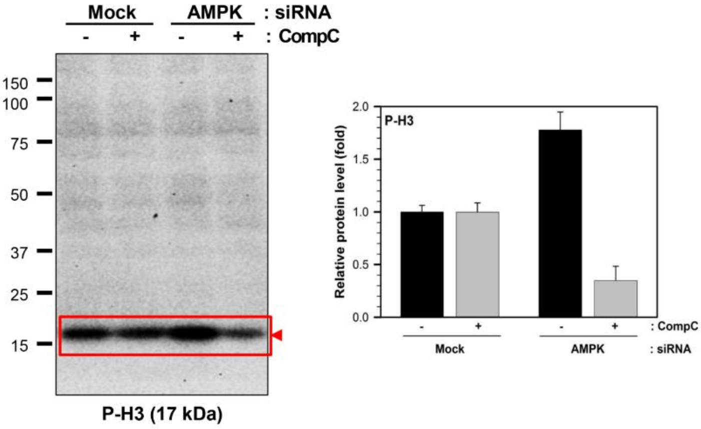

S2-5

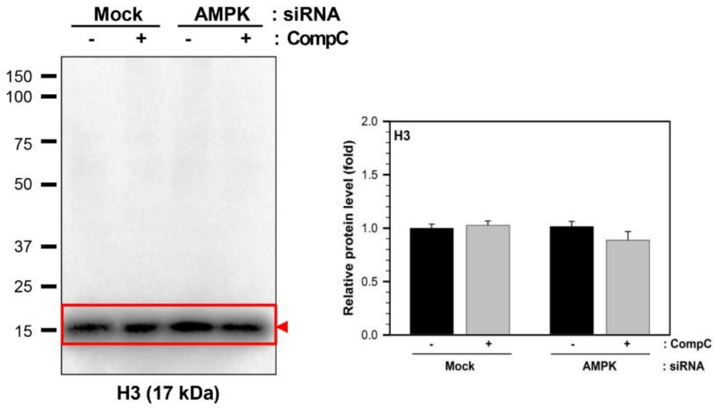

S2-6

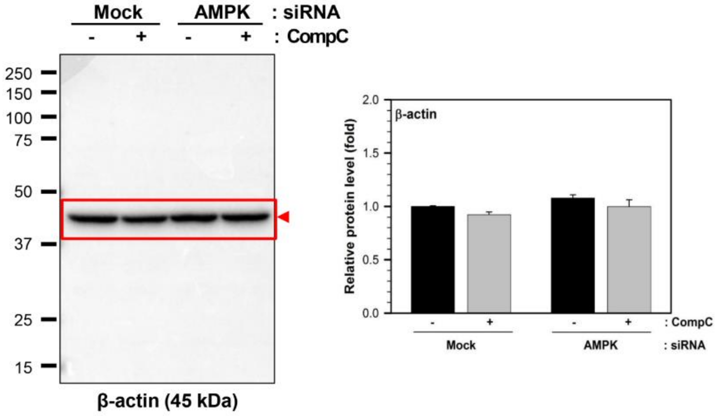

Figure S2. B16-F1 cells were transfected with mock or AMPKα1/2 siRNA for 48 h and then stimulated with 10% FBS in the presence of vehicle (−) or 10 μM CompC (+) for 16 h. Cell lysates were analyzed by western blot analysis using antibodies against AMPKα1/2, total and phosphorylated Cdc2, histone H3, and β-actin. The band density of β-actin was used as a normalization control for densitometry analysis of each blot. A representative whole blot and a graph with error bars for western blot analysis of each protein are shown side by side. (supplementary data for Figure 2C)

S3-1

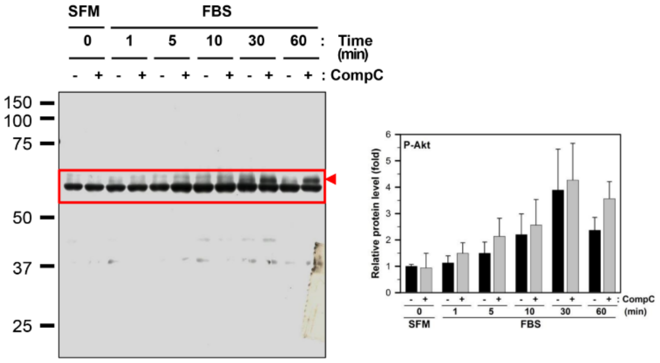

P-Akt (60 kDa)

S3-2

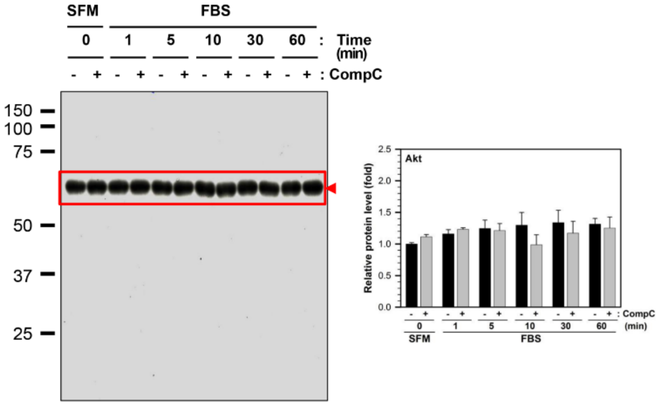

Akt (60 kDa)

S3-3

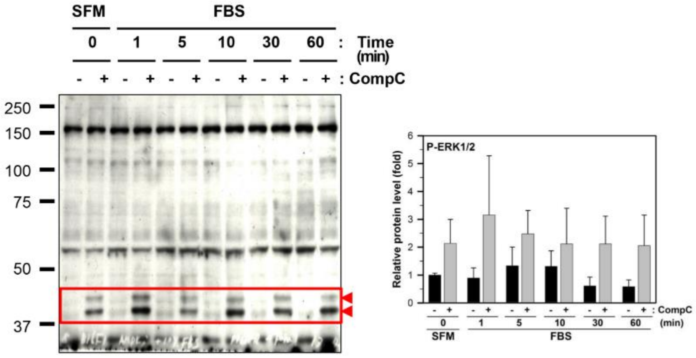

P-ERK1/2 (42/44 kDa)

S3-4

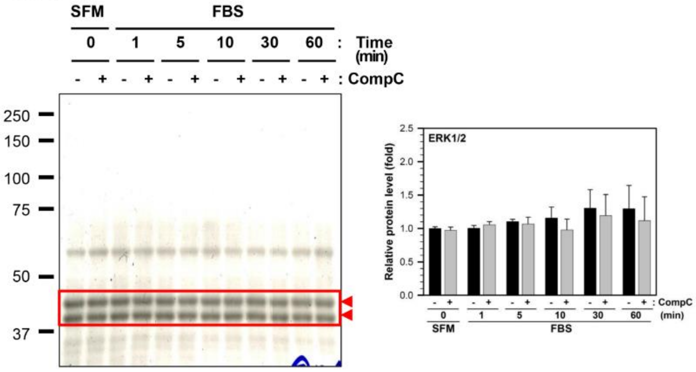

ERK1/2 (42/44 kDa)

S3-5

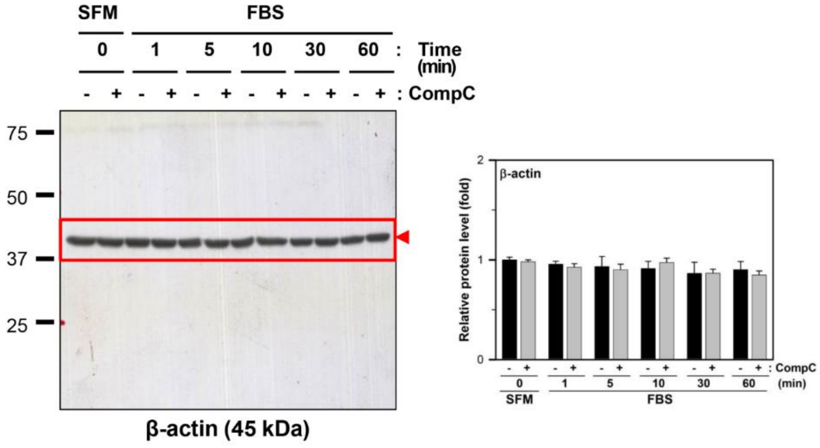

Figure S3. B16-F1 cells were stimulated with 10% FBS for the indicated times in the presence of vehicle or 10 μM CompC. Cell lysates were analyzed by western blot analysis using antibodies against total and phosphorylated Akt (P-Akt) and ERK1/2 (P-ERK1/2), and β-actin. The band density of β-actin was used as a normalization control for densitometry analysis of each blot. A representative whole blot and a graph with error bars for western blot analysis of each protein are shown side by side. (supplementary data for Figure 3A)

S4-1

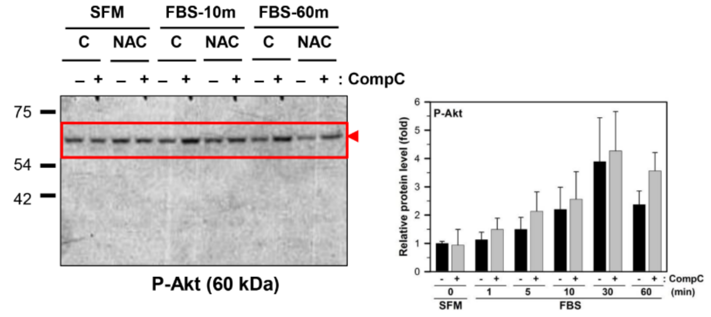

S4-2

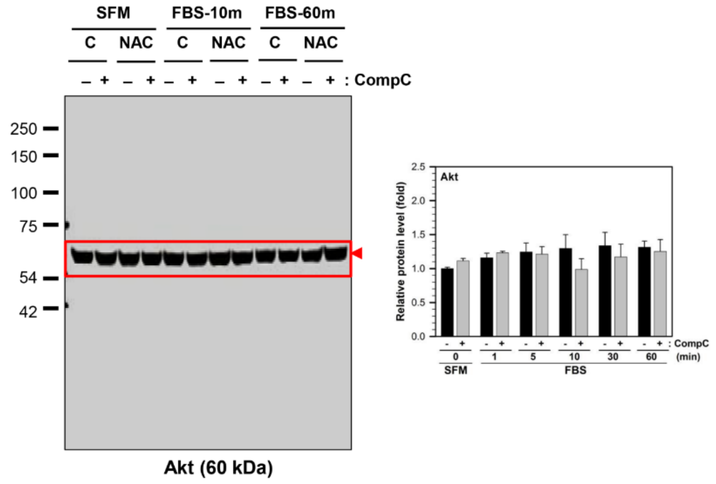

S4-3

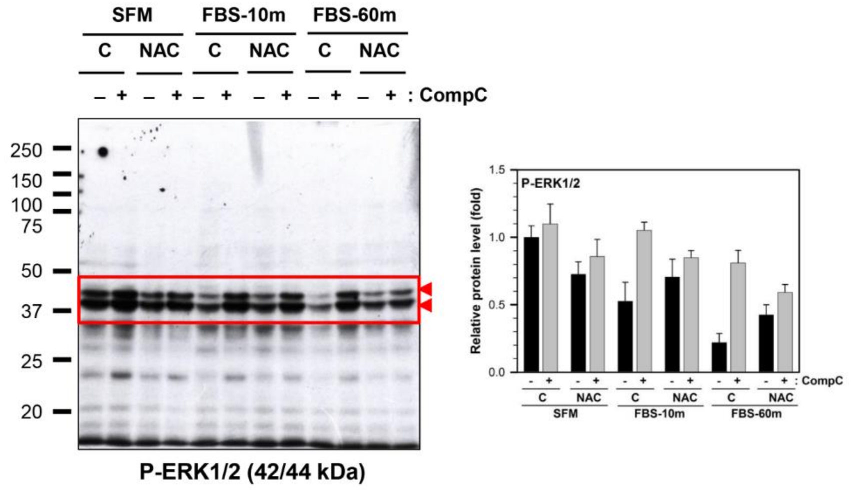

S4-4

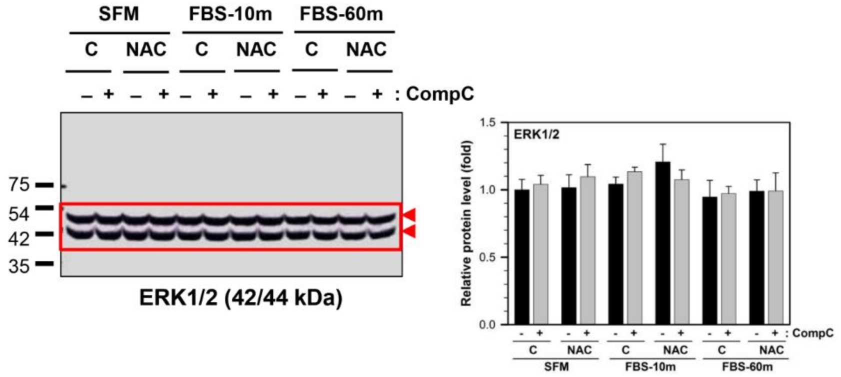

S4-5

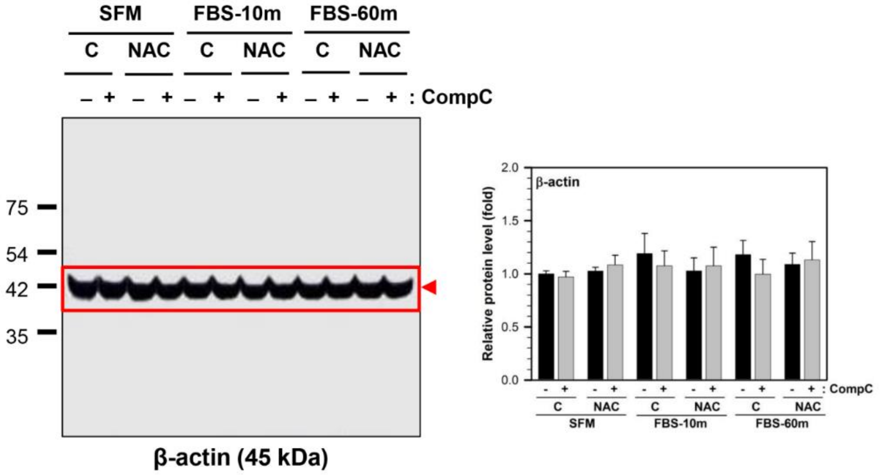

Figure S4. B16-F1 cells were serum-starved by incubation with SFM for 24 h. Cells were pretreated with vehicle alone, 5 mM NAC and/or 10 μM CompC for 1 h. Cells were then stimulated with FBS for 10 and 60 min. Cell lysates were analyzed by western blot analysis using antibodies against total and phosphorylated Akt (P-Akt) and ERK1/2 (P-ERK1/2), and β-actin. The band density of β-actin was used as a normalization control for densitometry analysis of each blot. A representative whole blot and a graph with error bars for western blot analysis of each protein are shown side by side. (supplementary data for Figure 3B)

S5-1

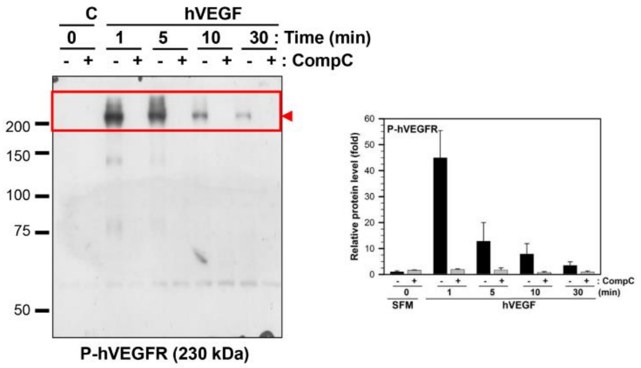

S5-2

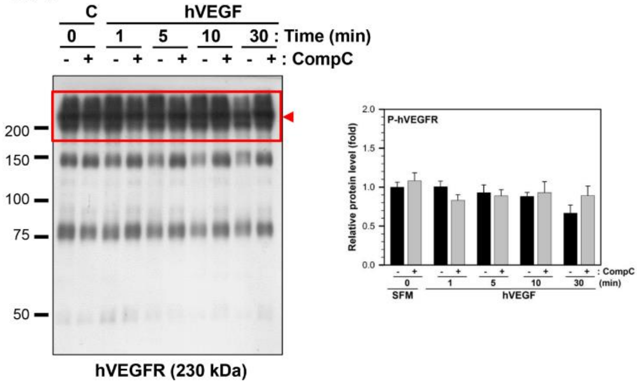

S5-3

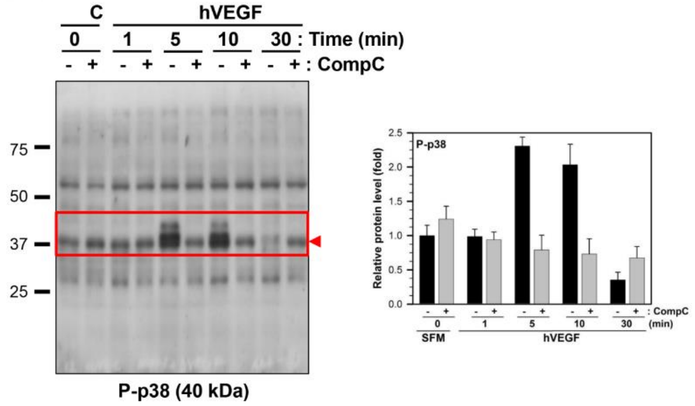

S5-4

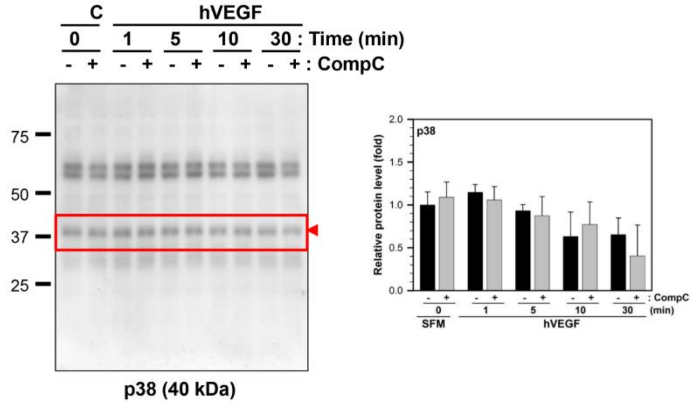

S5-5

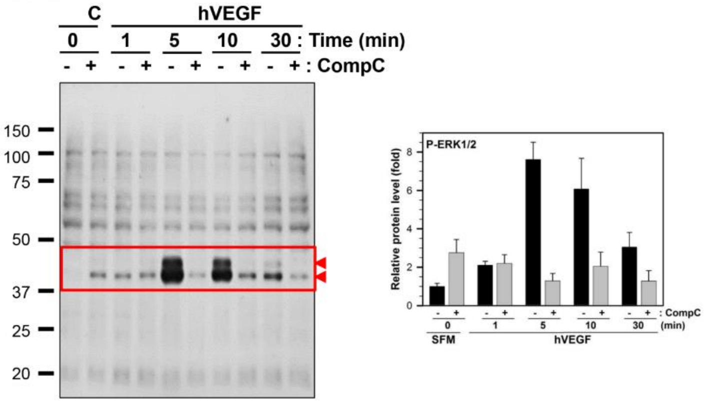

P-ERK1/2 (42/44 kDa)

S5-6

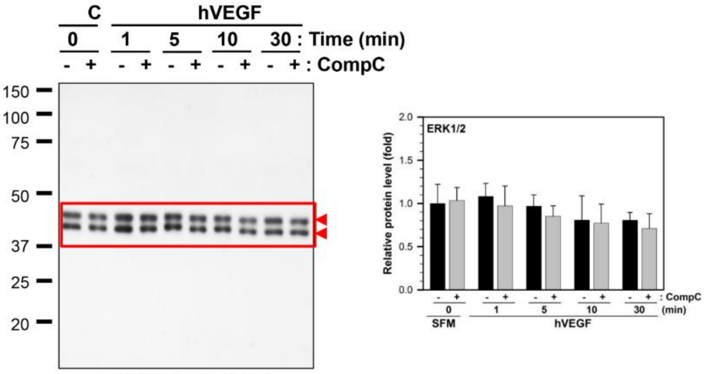

ERK1/2 (42/44 kDa)

S5-7

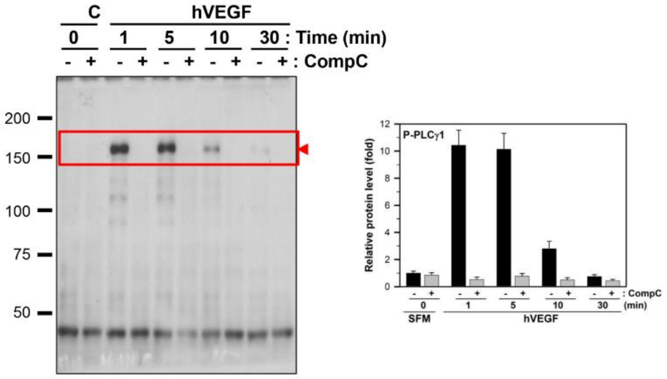

P-PLCγ1 (150 kDa)

S5-8

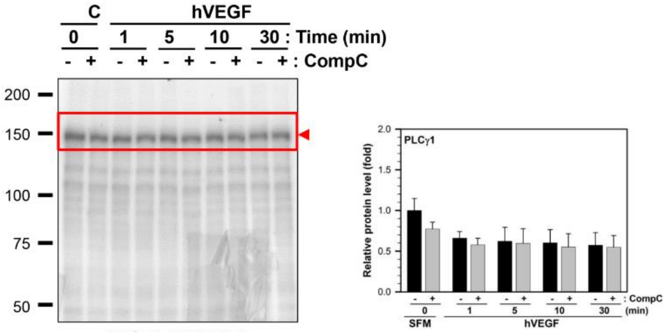

PLCγ1 (150 kDa)

S5-9

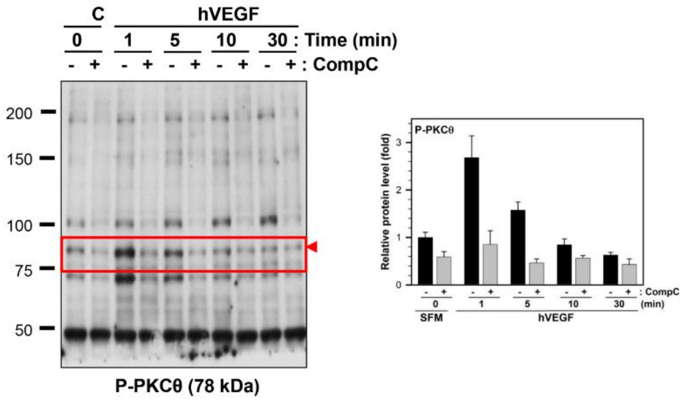

S5-10

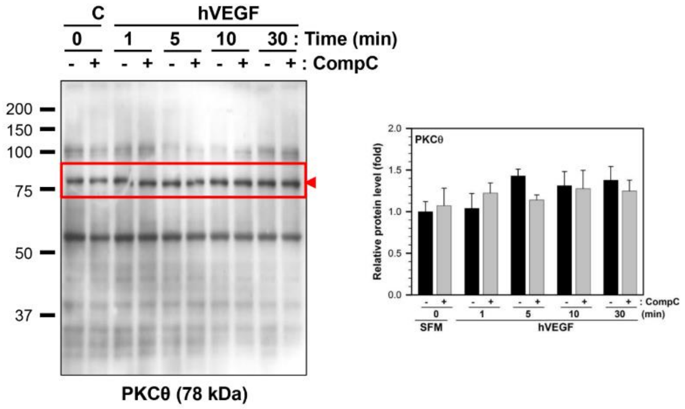

S5-11

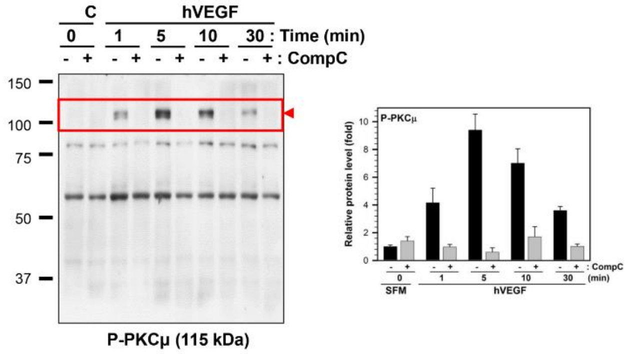

S5-12

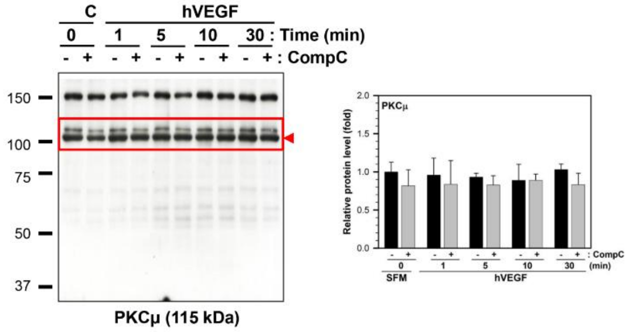

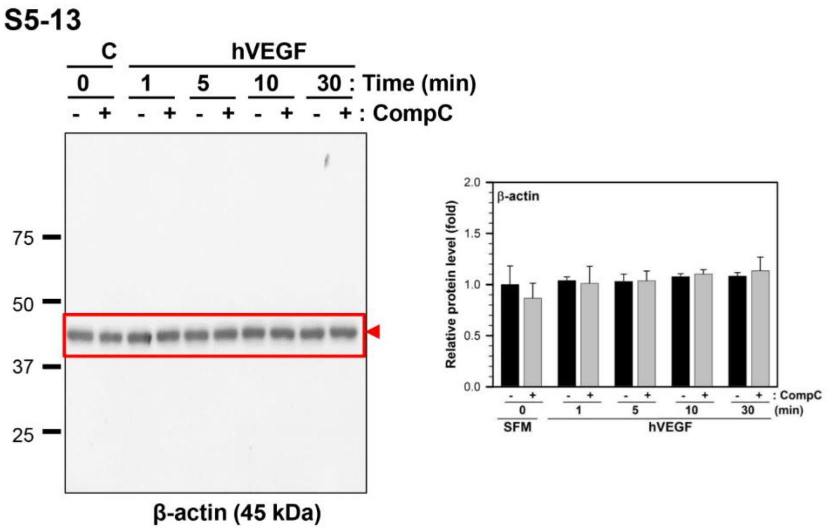

Figure S5. HUVECs were serum-starved by incubation with EBM for 24 h. Cells were treated with EBM containing 50 ng/ml hVEGF for the indicated times in the presence of vehicle (−) or 10 μM CompC (+). Cell lysates were analyzed by western blotting with antibodies against total and phosphorylated hVEGFR and other signaling proteins. The band density of β-actin was used as a normalization control for densitometry analysis of each blot. A representative whole blot and a graph with error bars for western blot analysis of each protein are shown side by side. (supplementary data for Figure 4F)

© 2019 by the authors. Licensee MDPI, Basel, Switzerland. This article is an open access article distributed under the terms and conditions of the Creative Commons Attribution (CC BY) license (http://creativecommons.org/licenses/by/4.0/).
